# The impact of an online, lifestyle intervention programme on the lives of patients with a rheumatic and musculoskeletal disease: a pilot study

**DOI:** 10.1093/rheumatology/keae696

**Published:** 2024-12-28

**Authors:** Kim van Slingerland, Laura J C Kranenburg, Nathalie Wilmsen, Emma Coles, Radboud J E M Dolhain, Pascal H P de Jong

**Affiliations:** Department of Rheumatology, Erasmus MC, Rotterdam, The Netherlands; Department of Rheumatology, Erasmus MC, Rotterdam, The Netherlands; Voeding Leeft, Amsterdam, The Netherlands; Voeding Leeft, Amsterdam, The Netherlands; Department of Rheumatology, Erasmus MC, Rotterdam, The Netherlands; Department of Rheumatology, Erasmus MC, Rotterdam, The Netherlands

**Keywords:** online lifestyle intervention programme, health risk, disease impact, patient-reported outcome measurements, inflammatory arthritis, osteoarthritis, fibromyalgia

## Abstract

**Objectives:**

To evaluate the short- and long-term effects of an online, interactive, multifactorial lifestyle intervention programme (Leef! Met Reuma) on health risk and all ICHOM-recommended patient-reported outcome measures (PROMs) in patients with an inflammatory arthritis (IA), osteoarthritis (OA) or fibromyalgia (FM).

**Methods:**

Patients with an IA, OA or FM could register for the lifestyle intervention programme. The programme consists of a 3-month intensive part followed by a 21-month aftercare period and focuses on four pillars, namely nutrition, exercise, relaxation and sleep. Health risk and PROMs are collected 3-monthly during the first 6 months and 6-monthly during the next 18 months. Health risk includes self-reported weight, waist circumference and BMI. Following PROMs were included: pain, morning stiffness severity, fatigue, Health Assessment Questionnaire, quality of life, perceived stress, sleep disturbance and impact on life. Descriptive statistics were used to assess the change in health risk and PROMs during the intensive part of the programme and aftercare period.

**Results:**

Of the 264 patients studied, 88, 105 and 71 were diagnosed with IA, OA and FM, respectively. Health risk significantly improved in all three diagnosis groups during the intensive part of the programme. The mean BMI reduction was −1.36 (0.26), −1.22 (0.23) and −1.48 (0.33), whereafter it stabilized in the aftercare period. All PROMs showed a similar trend.

**Conclusion:**

An online, interactive lifestyle intervention programme has a positive long-term effect, even after 2 years of follow-up, on health risk and all PRO domains in patients with an IA, OA and FM.

Rheumatology key messages

- Rheumatic and musculoskeletal diseases (RMDs) often have a major impact on patients’ lives, and can be exacerbated by an unhealthy lifestyle, inactivity and obesity.
- An online, interactive multifactorial lifestyle intervention programme shows a positive long-term effect on health risks and patient-reported outcome domains in patients with a RMD.
- The disease burden of patients with an RMD can be reduced by an online, interactive multifactorial lifestyle intervention programme, however, further research on the effect of the lifestyle intervention on disease activity in IA patients is needed.

## Introduction

Healthcare is shifting towards a person-centred care (PCC) approach, which is specifically recommended for patients with chronic conditions, including rheumatic and musculoskeletal diseases (RMDs) [1, 2]. In PCC, not only the disease but also its impact on patients’ lives is managed [3]. Although treatment is available for most of the RMDs, a majority of patients still report relevant disease symptoms even after achieving low disease activity or remission. These (persistent) disease symptoms, i.e. fatigue, pain and morning stiffness, often have a significant impact on the lives of RMD patients [4–7]. Addressing the disease impact during outpatient consultations places high demands on the healthcare system because of the aging population, global epidemiological transition and shortage of healthcare providers. One of the solutions for aforementioned problems might be the promotion of a healthy lifestyle in addition to standard care [8].

A healthy lifestyle, including the pursuit of a healthy weight, has a positive effect on the prevention and progression of chronic diseases and is, therefore, promoted by the World Health Organization (WHO) and various governments [9–11]. Not following this advice has a negative effect on the perceived disease impact in RMD patients [12]. Therefore, the recommendations of the European Alliance of Associations for Rheumatology (EULAR) state that WHO’s healthy lifestyle recommendations are also applicable to RMD patients and are an essential part of RMD management, complementary to the medical treatment [13].

A lifestyle intervention programme called ‘Plant for Joints’ in patients with rheumatoid arthritis and osteoarthritis already showed persistent positive effects up to one year after the intervention, such as improved metabolic status, stiffness, pain and function and disease activity in rheumatoid arthritis patients [14–16]. Nevertheless, the interventions are often labour intensive and time-consuming for the patient and the healthcare provider. Plant-based diets, which were used in aforementioned lifestyle interventions, are often difficult to maintain [17]. Most lifestyle intervention studies have a short follow-up, which makes proper evaluation of a sustained behavioural lifestyle change cumbersome. Finally, there are few data on the effect of the lifestyle intervention on reducing the disease impact and the impact of RMDs on various life domains [14–16].

To measure disease impact patient reported outcomes measurements can be used (PROMs) [18, 19]. The International Consortium for Health Outcomes Measurement (ICHOM) recently agreed on the most relevant PRO domains for IA and OA patients. Overlapping domains are pain, fatigue, activity limitation, overall emotional and physical health impact, work/school/housework ability and productivity [20]. None of the aforementioned lifestyle intervention studies took all ICHOM-recommend outcome domains into account [14–16].

Therefore, our aim is to investigate the short- and long-term effects of an online, interactive, multifactorial lifestyle intervention programme on health risks and all ICHOM-recommended PRO domains in patients with inflammatory arthritis (IA), osteoarthritis (OA) and fibromyalgia (FM).

## Methods

### Patients

A prospective cohort study was used to evaluate the effect of an online, interactive, multifactorial lifestyle intervention programme on the lives of patients with IA (including rheumatoid arthritis, psoriatic arthritis, spondyloarthritis, juvenile idiopathic arthritis), OA and FM. The study was approved by the Medical Ethics Committee of the Erasmus Medical Center and considered not subject to Dutch law and provided a waiver (MEC-2020-0729). This study was conducted in accordance with the principles of the Declaration of Helsinki.

Patients with IA, OA or FM could register for this study through the website of the Dutch Foundation Voeding Leeft. The diagnosis was verified by the treating rheumatologist. Other inclusion criteria were: (1) age ≥18 and ≤80 years; (2) body mass index (BMI) ≥18.5 and ≤35; (3) being motivated to work on a healthy lifestyle; (4) having a computer or tablet and an e-mail address and be able to communicate with it; and (5) being able to understand, speak and write Dutch.

Exclusion criteria were: (1) having another (chronic) condition, such as Crohn’s disease/ulcerative colitis, cardiovascular disease, cancer, mental health problems and/or addictions; (2) women who were pregnant or lactating; (3) having a history of bariatric surgery; (4) having (a history of) an eating disorder; and (5) participating (or previous participated) in another lifestyle programme or study.

### Study design

A 24-month prospective cohort study was set up in which all patients followed an online, interactive, multifactorial lifestyle intervention programme, called ‘Leef! Met Reuma’ (developed by the Dutch foundation Voeding Leeft). The programme consisted of an intensive part of 3 months, followed by a 21 months aftercare period.

The lifestyle programme focuses on four pillars: nutrition, exercise, relaxation and sleep. The prescribed diet is comparable to the Mediterranean diet, with an emphasis on unprocessed foods (especially vegetables). Patients are encouraged to consume three meals a day (no snacking) and avoid alcohol consumption. The lifestyle intervention focuses on a sustainable behaviour change (based on the I-Change model) [21]. The guidance team consists of a (lifestyle) coach, dietician, programme coordinator and experts in physical activity, sleep and relaxation.

During the intensive part, patients followed three plenary online meetings of 5 h each, in groups of 100 patients, at baseline, 1 and 3 months, and six coaching sessions in smaller groups of 25–33 patients. In the aftercare period, patients had the opportunity to attend optional follow-up meetings on the four pillars to maintain their behavioural change. All meetings were given via Zoom. Patients also had access to a secure online platform during the 24-month programme where they could find more background information, ask questions (to the programme coordinator, nutritionist, (lifestyle) coach or peers) and/or share their experiences. They could also track their progress in a personalized dashboard, find recipes/grocery lists and explore additional content such as podcasts.

### Data collection

Patients filled out online questionnaires at baseline and after 3, 6, 12, 18 and 24 months. At each time point weight, waist circumference, PROMs, programme satisfaction and adherence difficulty were collected. Length and demographics were collected at baseline. The following PROMs were collected: pain, morning stiffness severity, fatigue, activity limitations, (health-related) quality of life, perceived stress, sleep disturbance and impact on work productivity/life. To measure the effect of the lifestyle intervention, the overlapping ICHOM PRO domains were collected in all three diseases and we choose PROMs that have been used/validated in all three diseases in previous literature.

Pain and fatigue were measured with a Visual Analogue Score (VAS), ranging from 0 to 100 and morning stiffness severity with an 11-point (0–10) Numeric Rating Scale (NRS) retrieved from the Bath Ankylosing Spondylitis Disease Activity Index questionnaire [22]. In these three PROMs, a lower score indicates better outcomes.

Activity limitations were measured with the validated Dutch version of the Health Assessment Questionnaire (HAQ) and includes 20 specific functions that are grouped into 8 categories, namely dressing and grooming, arising, eating, walking, personal hygiene, reaching, gripping and other activities [23]. The statements can be scored by a 4-point Likert scale, from no difficulty (0) to unable to do (3), and a higher score corresponds with greater functional impairment.

Quality of life was measured with the European quality of life with-5 dimensions with 5 levels (EQ-5D-5L) and the 36-item Short Form Health Survey (SF-36). The EQ-5D-5L scores quality of life on five health dimensions, namely mobility, self-care, daily activities, pain/discomfort and anxiety/depression, which are answered with a 5-point Likert scale. The calculated score ranged from 0 to 1; 0 equals death and 1 equals perfect health [24]. The SF-36 covers the following eight domains: physical functioning, bodily pain, role limitations due to physical health problems and personal or emotional problems, general mental health, social functioning, energy/fatigue and general health perceptions. For each domain, item scores were summed into scale scores and transformed to a 100-point scale, with higher scores reflecting better health-related quality of life. These scores were compared with the general population [25, 26].

Perceived stress was measured with the Perceived Stress Scale (PSS) and contains 10 questions answered with a 5-point Likert scale, ranging from ‘I never had these feelings/thoughts the last month’ (0) to ‘I had these feelings/thoughts very often the lost month’ (4). The sum score ranges from 0 to 40 and can be divided into following categories: low stress (0–13), moderate stress (14–26) and high stress (27–40) [27].

Sleep disturbance was measured with the Sleep Scale from the Medical Outcomes Study (MOS-ss) [28]. The 12 items can be scored with a 6-point Likert Scale, from ‘all of the time’ (1) to ‘none of the time’ (6). Each item was transformed to an achieved percentage of the total possible score and was used to calculate the Sleep Problems Index II (SPI-II). Higher scores corresponds with more sleep problems [28].

The impact of the disease on work ability and life was measured with the Work Productivity and Activity Impairment (WPAI) questionnaire and were assessed with a 11-point Likert scale, ranging from ‘no effect’ (0) to ‘completely prevented me’ (10). Higher scores correspond with more impact on work ability and life [29].

Programme satisfaction and difficulty were measured with an 11-point (0–10) NRS and higher scores indicates higher levels of satisfaction and adherence difficulty, respectively.

### Statistical analysis

Descriptive statistics were used to assess the change in health risks and PROMs during the intensive part and the follow-up period. A paired *t* test was used to examine whether the improvement in health risks and PROMs during the intensive part was significant. Hereafter, we tested whether the improvements achieved in health risks and PROMs did not deteriorate during the follow-up period by looking at the difference between outcomes at 3 and 24 months. PROM changes of IA patients were compared with the minimal clinically important difference (MCID) to determine whether the improvements or deteriorations were clinically relevant [30]. The MCIDs of the different PROMs are shown in Supplementary Table S1, available at *Rheumatology* online [31–37].

Because of drop-out ratios above 10%, we handled missing data with multiple imputations with chained equations (MICE) with 40 imputations [38]. Missing data were imputed for weight, BMI, waist circumference, pain, morning stiffness severity, fatigue, HAQ, EQ-5D-5L, SF-36 domains, perceived stress, sleep disturbance, impact work ability, impact on life and the different facets of programme satisfaction and difficulties. In the imputation regression models, diagnosis, time point, age and sex were the independent variables.

In addition, two sensitivity analyses were performed. First, the reason for drop-out might not be at random and, therefore, a complete case analysis was conducted. A complete case was defined as having a self-reported weight, BMI and waist circumference at all time-points. Secondly, participants with ≥5% weight loss after the intensive part of the lifestyle intervention programme were analyzed, because we assumed that this weight loss was associated with the greatest health benefits and programme compliance.

To correct for multiple testing, a Bonferroni correction was applied by multiplying the *P*-values with 30, because of the 10 performed test in each diagnosis group (IA, OA and FM) [39]. A corrected *P*-value < 0.05 was considered statistically significant. All analyses were performed in Stata version 18.

## Results

### Baseline outcomes

The baseline characteristics of the 264 participants are shown in Table 1. A total of 88 patients were diagnosed with IA, 105 with OA and 71 with FM. The mean age (standard deviation, SD) was 52 (12) years and 93% was female. The mean (SD) BMI was 28 (4), 26 (5) and 28 (4) for IA, OA and FM, respectively. Most of the patients had a low educational level which applied for all diagnosis groups (65% in IA, 66% in OA and 48% in FM).

**Table 1.** Baseline characteristics

	IA (*n* = 88)	OA (*n* = 105)	FM (*n* = 71)
Sex, female, *n* (%)	78 (92.9)	80 (89.9)	66 (97.1)
Age, year, mean (SD)	58.7 (9.1)	49.5 (10.7)	47.3 (12.2)
Nationality: Dutch, *n* (%)	76 (88.4)	93 (88.6)	66 (93.0)
Educational level^a^: low, *n* (%)	56 (65.1)	69 (65.7)	34 (47.9)
Intermediate, *n* (%)	14 (16.3)	10 (9.5)	7 (9.9)
High, *n* (%)	16 (18.6)	26 (24.8)	30 (42.3)
Weight, kg, mean (SD)	80.5 (13.5)	78.2 (14.1)	80.2 (14.2)
BMI, kg/m^2^, mean (SD)	28.0 (4.3)	26.4 (4.6)	27.6 (4.4)
Waist circumference, cm, mean (SD)	96.7 (12.0)	92.6 (12.4)	96.1 (13.5)

a Educational level: low corresponds to primary school, lower and intermediate secondary schooling or intermediate vocational training; Intermediate corresponds to higher secondary schooling or intermediate vocational training; and high corresponds to higher vocational training or university.

Abbreviations: BMI, body mass index; cm, centimetre; FM, fibromyalgia; IA, inflammatory arthritis, including rheumatoid arthritis, psoriatic arthritis, spondylarthritis or juvenile idiopathic arthritis; kg, kilogram; OA, osteoarthritis; SD, standard deviation.

Drop-out rates within the IA, OA and FM group were 28% (25/88), 33% (35/105) and 51% (36/71) after 1 year and increased to 33% (29/88), 41% (43/105) and 52% (37/71) after 2 years of follow-up, respectively (Supplementary Fig. S1, available at *Rheumatology* online). Main reasons for drop-out were too time consuming and loss of interest.

### Health risk over time

**Figure 1.**
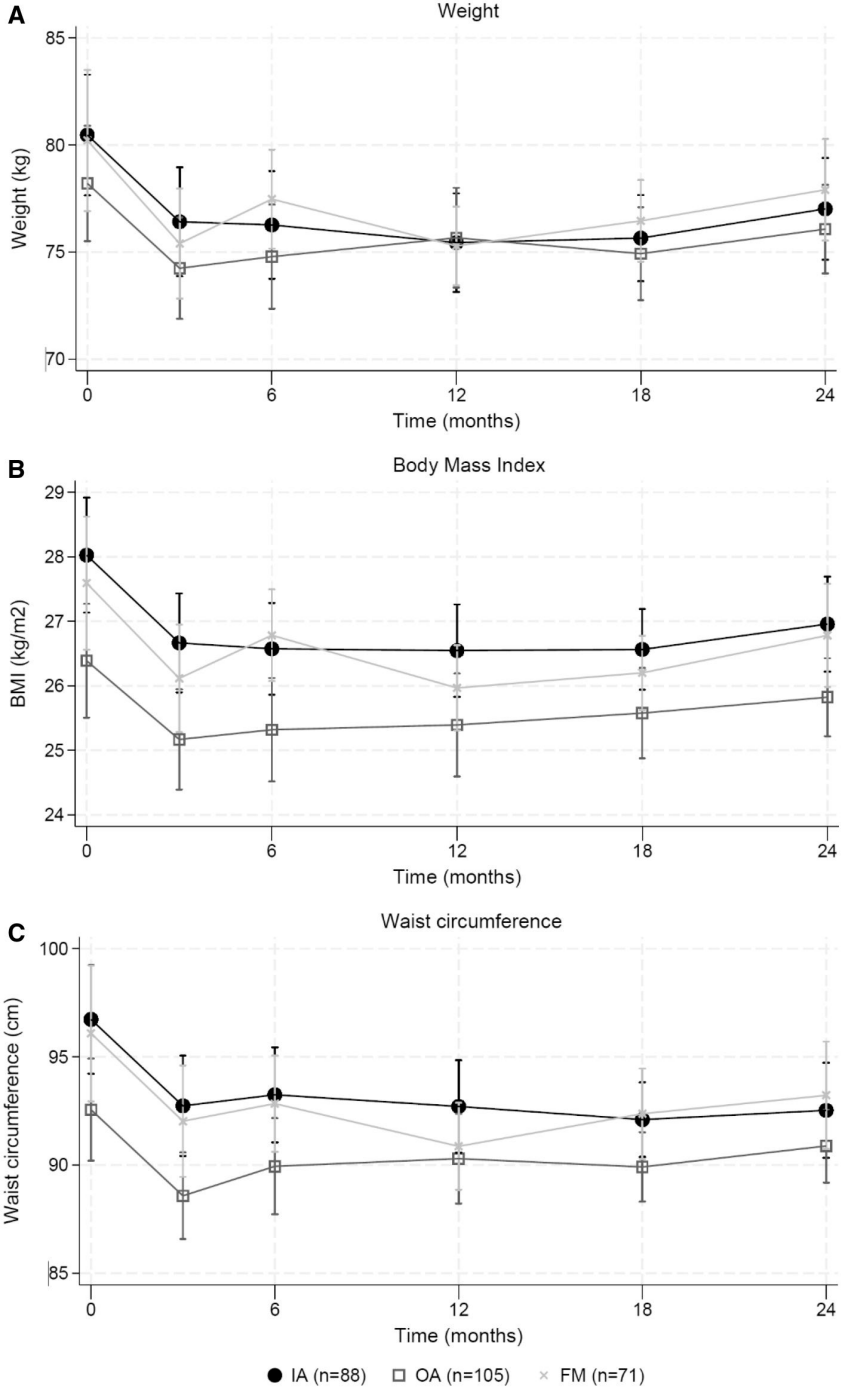
Health risk over time stratified per diagnose group. (**A**) Weight, (**B**) Body Mass Index and (**C**) Waist circumference. Abbreviations: BMI, body mass index; cm, centimetre; FM, fibromyalgia; IA, inflammatory arthritis, including rheumatoid arthritis, psoriatic arthritis, spondyloarthritis or juvenile idiopathic arthritis; kg, kilogram; OA, osteoarthritis

The changes in mean weight, BMI and waist circumference over time stratified for diagnosis are shown in Fig. 1. In the intensive part of the intervention the mean (SD) weight reduction was −4.2 (0.7), −3.9 (0.7) and −4.3 (1.0) kg, while in the aftercare period, the mean (SD) weight slightly increased again with 0.7 (0.8), 1.7 (0.8) and 2.5 (1.2) kg in the IA, OA and FM group, respectively. Similar changes were found for BMI and waist circumference. The improvement in health risk during the first 3 months was statistically significant in all three patient groups. For the total intervention period, only weight loss and waist circumference reduction in IA patients were observed (Table 2).

**Table 2.** Health risk and PROM changes during the intensive part and aftercare period of the online, interactive, multifactorial lifestyle intervention programme

	IA (*n* = 88)			OA (*n* = 105)			FM (*n* = 71)		
	**0–3 months** *	**3–24 months** *	**0–24 months** *	**0–3 months** *	**3–24 months** *	**0–24 months** *	**0–3 months** *	**3–24 months** *	**0–24 months** *
Health risks									
Weight (kg)	−4.2 (0,7)*	0.7 (0.8)	−3.5 (1.0)^⁰^	−4.0 (0.7)*	1.7 (0.8)	−2.2 (1.0)	−4.3 (1.0)*	2.5 (1.2)	−1.8 (1.3)
BMI (kg/m^2^)	−1.4 (0.3)*	0.3(0.2)	−1.1 (0.3)	−1.2 (0.2)*	0.7 (0.3)	−0.6 (0.3)	−1.5 (0.3)*	0.7 (0.3)	−0.8 (0.4)
Waist circumference (cm)	−3.9 (0.7)*	−0.1 (0.8)	−4.0 (1.0)^i^	−4.0 (0.8)*	2.1 (0.8)	−1.9 (1.0)	−3.7 (1.0)^i^	1.2 (1.1)	−2.5 (1.2)
PROMs									
Pain (VAS 0–100)	−5 (2)	−3 (2)	−8 (3)^⁰^	−4 (2)	1 (2)	−3 (3)	−9 (3)	−1 (3)	−10 (3)^⁰^
MS severity (NRS 0–10)	−**1(0**^i^)	−0(0)	−**1(0)**^⁰^	−1(0)^⁰^	−0(0)	−1(0)^⁰^	−1(0)*	−0(0)	−1(0)*
Fatigue (VAS 0–100)	−6 (3)	2 (3)	−4 (3)	−14 (3)*	1 (2)	−12 (3)*	−11 (4)	2 (3)	−9 (3)
HAQ (0–3)	−0.1 (0.0)	−0.0 (0.1)	−0.1 (0.1)	−0.1 (0.0)	0.0 (0.0)	−0.1 (0.0)	−0.0 (0.0)	−0.1 (0.1)	−0.1 (0.1)
EQ-5D-5L (0–1)	**0.0 (0.0)**	0.0 (0.0)	0.0 (0.0)	0.1 (0.0)*	0.0 (0.0)	0.1 (0.0)*	0.1 (0.0)	0.0 (0.0)	0.1 (0.0)^⁰^
Perceived stress (PSS 0–40)	−1.2 (0.5)	0.5 (0.5)	−0.7 (0.7)	−1.8 (0.5)^⁰^	−1.0 (0.5)	−2.8 (0.6)*	−2.5 (0.6)^i^	0.1 (0.7)	−2.4 (0.7)^⁰^
Sleep disturbance (SPI-II 0–100)	−6.1 (1.4)*	0.2 (1.5)	−5.9 (1.8)^⁰^	−4.9 (1.4)^⁰^	0.0 (1.5)	−4.4 (1.6)	−4.8 (1.8)	0.9 (1.7)	−5.7 (1.9)
Impact on life (NRS 0–10)	−0.4 (0.3)	−0.3 (0.3)	−0.7 (0.3)	−0.7 (0.3)	0.1 (0.3)	−0.5 (0.3)	−1.1 (0.3)^i^	−0.2 (0.3)	−1.3 (0.3)*

* Statistical comparisons were performed between baseline and 3 months, 3 and 24 months, and baseline and 24 months, using a paired *t* test.

For each variable mean (SD) and statistical significance are reported.

⁰ *P* < 0.05.

i *P* < 0.01.

* *P* < 0,001.

Clinically relevant improvements compared with MCID are shown in bold.

Abbreviations: BMI, body mass index; cm, centimetre; EQ-5D-5L, European quality of life with-5 dimensions with 5 levels; FM, A; HAQ, Health Assessment Questionnaire; IA, inflammatory arthritis, including rheumatoid arthritis, psoriatic arthritis, spondyloarthritis or juvenile idiopathic arthritis; kg, kilogram; MCID, minimal clinical important difference; MS, morning stiffness; NRS, Numeric Rating Scale; OA, osteoarthritis; PROM, patient-reported outcome measure; PSS, Perceived Stress Scale; SD, standard deviation; SPI-II, Sleep Problems Index II; VAS, Visual Analogue Score.

### PROMs over time

**Figure 2.**
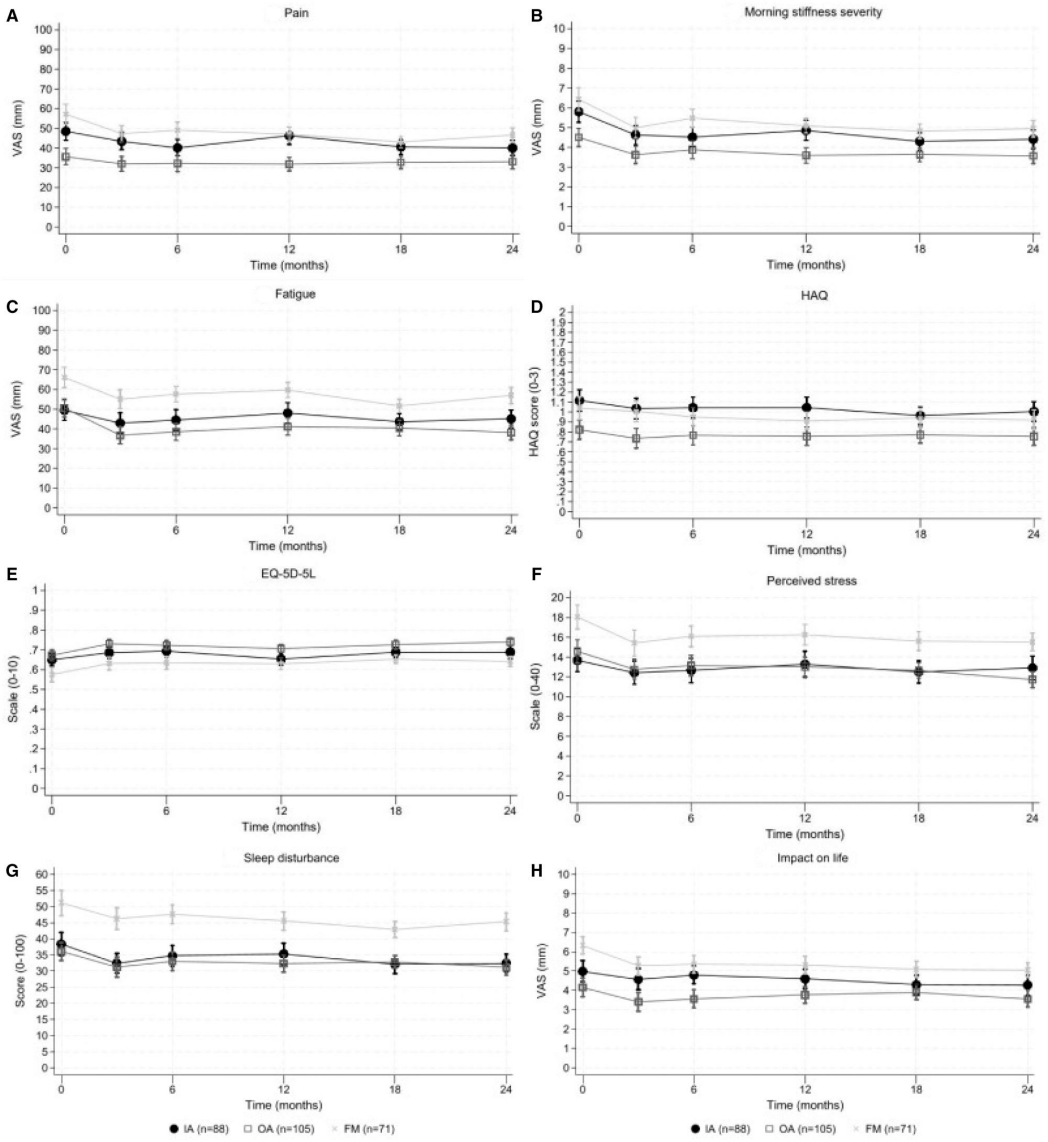
PROMs over time stratified per diagnose group. (**A**) Pain, (**B**) Morning stiffness severity, (**C**) Fatigue, (**D**) HAQ, (**E**) EQ-5D-5L, (**F**) Perceived stress, (**G**) Sleep disturbance and (**H**) Impact on life. All figures show the mean with corresponding 95% confidence interval for the respective PROM. Abbreviations: EQ-5D-5L, European quality of life with 5 dimensions with 5 levels; FM, fibromyalgia; HAQ, Health Assessment Questionnaire; IA, inflammatory arthritis, including rheumatoid arthritis, psoriatic arthritis, spondyloarthritis or juvenile idiopathic arthritis; OA, osteoarthritis; PROMs, patient-reported outcome measure; VAS, Visual Analogue Score

The PROMs related to disease symptoms, such as pain, morning stiffness, and fatigue, improved the most during the intensive part, while they slightly worsened during the aftercare period, but they were still better than the baseline values. All other PROMs showed a similar trend (Fig. 2).

In IA patients morning stiffness severity (mean (SD) −1 (0), *P* = 0.003) and sleep disturbance (−6.1 (1.4), *P* = 0) significantly improved during the first 3 months of the intervention programme, these improvements also exceeded the MCID. Over 24 months, significant improvements were seen in IA patients for pain (−8 (3), *P* = 0.045), morning stiffness severity (−1 (0), *P* = 0.015) and sleep disturbance (−5.9 (1.8), *P* = 0.039). The MCID, however, was only exceeded for morning stiffness severity (Table 2).

In OA patients, significant PROM changes during the first 3 months of the intervention were found for morning stiffness severity (−10 (0), *P* = 0.042), fatigue (−14 (3), *P* = 0), EQ-5D-5L (0.1 (0.0), *P* = 0), perceived stress (−1.8 (0.5), *P* = 0.018) and sleep disturbance (−4.9 (1.4), *P* = 0.015) (Table 2). After 24 months, morning stiffness severity (−1 (0), *P* = 0.015), fatigue (−12 (3), *P* = 0), EQ-5D-5L (0.1 (0.0), *P* = 0) and perceived stress (−2.8 (0.6), *P* = 0) were all still significantly better compared with baseline.

In FM patients, significant PROM changes during the intensive part were found for morning stiffness severity (−1 (0), *P* = 0), perceived stress (−2.5 (0.6), *P* = 0.003) and impact on life (−1.1 (0.3), *P* = 0.003) (Table 2). Significant PROM changes for FM patients over 24 months were found for pain (−10 (3), *P* = 0.018), morning stiffness severity (−1 (0), *P* = 0), EQ-5D-5L (0.1 (0.0), *P* = 0.024), perceived stress (−2.4 (0.7), *P* = 0.021) and impact on life (−1.3 (0.3), *P* = 0).

**Figure 3.**
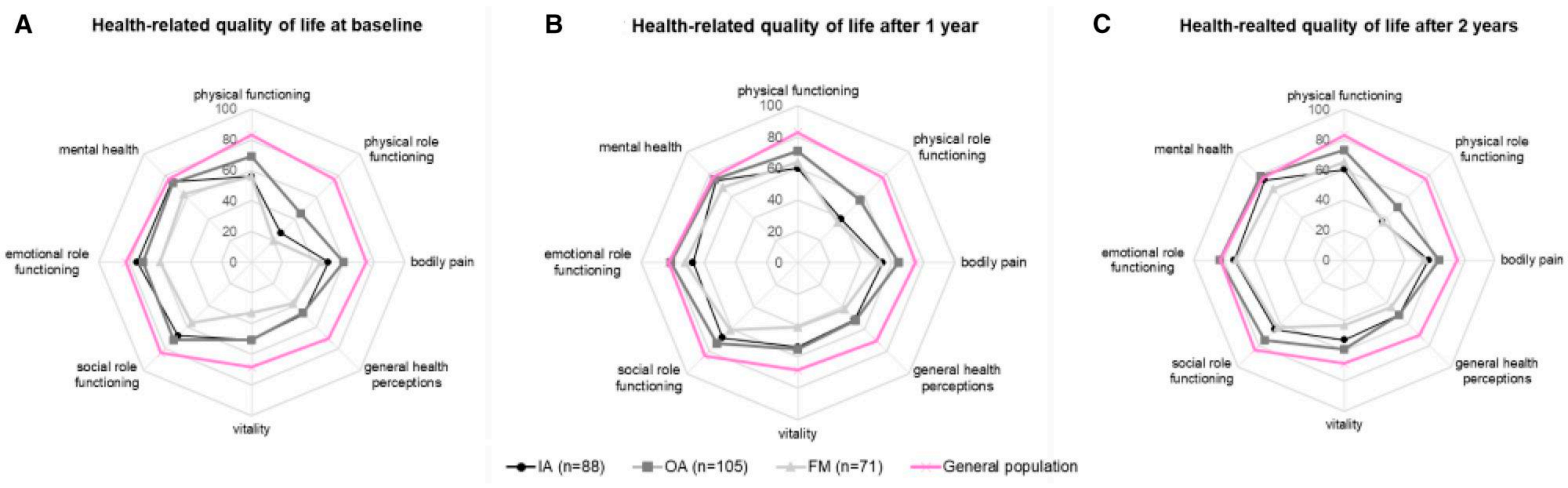
Health-related quality of life. (A) Mean HRQoL at baseline, (B) after 1 year, and (C) after 2 years, measured with the short form-36, stratified for IA, OA and FM, and compared to the Dutch general population norms. Abbreviations: FM, fibromyalgia; HRQoL, health-related quality of life; IA, inflammatory arthritis, including rheumatoid arthritis, psoriatic arthritis, spondyloarthritis or juvenile idiopathic arthritis; OA, osteoarthritis

We also examined the eight SF-36 domains at baseline, after 1 and 2 years and compared this with the general Dutch population. At baseline, the physical HRQoL domains, i.e. physical functioning, physical role functioning, bodily pain and vitality, were lower compared with the Dutch general population, but these domains also improved the most over time (Fig. 3). Of the emotional HRQoL domains, social role functioning was the most impaired and this domain did not improve over time.

### Programme satisfaction

All patient groups were generally satisfied with the lifestyle intervention programme (Table 3). The overall score was 7.1 (SD 1.3), with an 7.5 (SD 1.4) after the intensive part and 7.0 (SD 1.5) after the aftercare period. In terms of the different pillars, patients were most satisfied with the nutrition and physical activity pillar. These pillars scored 7.6 (SD 1.3) and 6.6 (SD 1.6), respectively. The overall difficulty of adhering to the principles/health advice of the intervention scored a 5.8 (SD 1.8), with a 5.1 (SD 2.2) after 3 months and a 6.1 (SD 1.8) after 24 months.

**Table 3.** Programme satisfaction, overall and per pillar, and difficulty of the online, interactive, multifactorial lifestyle intervention programme

	IA (*n* = 88)	OA (*n* = 105)	FM (*n* = 71)
Programme satisfaction			
• Overall	6.9 (1.5)	7.2 (1.3)	7.1 (1.1)
• 3 months	7.6 (1.6)	7.5 (1.3)	7.5 (1.2)
• 24 months	6.6 (1.6)	7.3 (1.5)	6.9 (1.1)
Satisfaction per pillar			
• Food	7.6 (1.4)	7.6 (1.4)	7.5 (1.0)
• Physical activity	6.9 (1.7)	6.6 (1.7)	6.4 (1.4)
• Sleep	6.2 (1.9)	6.0 (2.0)	6.3 (1.6)
• Relax	6.4 (1.7)	6.1 (1.7)	6.1 (1.4)
Difficulty			
• Overall	5.5 (1.9)	5.6 (1.9)	6.4 (1.6)
• 3 months	5.2 (2.2)	4.7 (2.1)	5.8 (2.2)
• 24 months	5.8 (2.1)	5.9 (1.9)	6.7 (1.2)

For each variable mean (SD) are reported.

Abbreviations: FM, fibromyalgia; IA, inflammatory arthritis, including rheumatoid arthritis, psoriatic arthritis, spondyloarthritis or juvenile idiopathic arthritis; OA, osteoarthritis.

### Sensitivity analysis

Two sensitivity analyses were performed, namely a complete case analysis and an analysis among patients with ≥5% weight loss after the intensive part of the lifestyle intervention programme. The results are presented in the Supplementary Material, available at *Rheumatology* online. Although the outcomes are less and more pronounced in the complete case and patients with ≥5% weight loss analysis, respectively, they all show a similar trend with improvement during the intensive part of the programme whereafter stabilization during the aftercare period.

## Discussion

We evaluated the effect of an online, interactive, multifactorial lifestyle intervention programme on health risks and all overlapping ICHOM PRO domains in patients with an IA, OA and FM. Health risks (weight, BMI and waist circumference) significantly improved in all groups during the intensive part of the programme, whereafter it stabilized in the aftercare period. The PROMs related to disease symptoms, such as pain, morning stiffness and fatigue, improved the most during the intensive part, thereafter the outcomes slightly worsened, but even after 2 years, these results remain better than at baseline. All other PROMs showed a similar trend. All results were more evident in patients with ≥5% weight loss during the intensive part. Patients themselves were relatively satisfied with the programme and found it moderately difficult to adhere to the programme principles.

Multidisciplinary lifestyle interventions studies that focus on different pillars, such as nutrition, exercise, relaxation and sleep, are scarce in the field of rheumatology. Recently, the ‘Plants for joints’ trials in patients with RA and OA were published, which showed similar results in improvements in health risk during the intervention period [14–16]. However, within the ‘Plants for joints’ trials, it is unclear whether these effects were long lasting and what the effect of the combined lifestyle intervention on all overlapping ICHOM recommended PROs was [14–16]. Our study indicates an improvement in disease impact up to 2 years and appears to produce long-term behavioural change. However, clinical outcomes, e.g. disease activity in IA, are lacking.

The most important differences between the ‘plant for joints’ and our lifestyle intervention programme are: (1) physical *vs* online meetings and (2) the type of diet. Our programme had several mandatory online meetings, which makes the programme less labour intensive for patients as well as the provider. Moreover, the prescribed diet, which resembles the Mediterranean diet with an emphasis on unprocessed foods, is easier to adhere to than a completely plant-based diet, because it’s less restrictive and more comparable to the Dutch diet [17]. Therefore, in our opinion, our lifestyle intervention programme is more accessible and easier to maintain in comparison to other programmes. However, this may be at the expense of the (clinical) outcomes.

For a lifestyle intervention programme to be successful, a sustainable behavioural change must be achieved. However, behaviour change is a difficult process and there are several theories that describe this [21]. The trans theoretical model, on which the I-change model is based, proposes five stages of behavioural change: precontemplation, contemplation, preparation, action and maintenance [40–42]. Based on this model, we can assume that patients following a lifestyle intervention programme are in the preparation, action and maintenance stages. It is normal for patients not to succeed immediately, because there are different stages of progression and regression through the stages of change [41]. Each relapse allows the patient to take the next action and learn from past mistakes [40]. This may explain the different success levels of patients who followed our lifestyle intervention programme. If patients are more intrinsically motivated, they are better able to change their behaviour [43]. Intrinsic motivation is determined by the level of autonomy, competence and connectedness [43]. These were at focus of our lifestyle intervention programme and may have indirectly contributed to the positive long-term effects.

To measure the effect of the lifestyle intervention on patients’ lives, PROMs were used. PROMs allow patients to self-report their outcomes in various life domains. A disadvantage of PROMs is that they are subjective and the timing of data collection can influence results [44]. Therefore, trials are needed that evaluate the clinical effectiveness, including disease activity and ability to safely taper medication, of a multidisciplinary lifestyle intervention programme in patients with RMDs, especially IA who represent the majority of patients who regularly visit the rheumatology outpatient clinic.

A limitation of this study was that no control group was included. Secondly, a relatively small number of patients were included and as a result IA patients, e.g. could not be divided into their specific diseases. However, almost all other lifestyle intervention studies have been conducted with smaller number of patients. Thirdly, the overlapping ICHOM recommended PRO domains for IA and OA patients were used for all patient groups. This was determined to properly assess the effect of the intervention for all patient groups. The chosen questionnaires within the ICHOM domains are generic, with the exception of the HAQ, although this questionnaire has also been used in OA and FM [45]. The use of various PROMs made multiple testing unavoidable. To avoid false-positive results, a Bonferroni correction was applied. In addition, the PROM changes of IA patients were compared with the MCID. Fourthly, a high drop-out rate due to not filling out the online questionnaires was observed, despite the fact that patients were able to self-enrol and thus probably had a high intrinsic motivation. Most patients dropped out in the FM group and at the end of follow-up. There may be various reasons for dropping out, such as ineffectiveness of the lifestyle intervention programme, loss of interest or too time-consuming. To account for missing data, we performed multiple imputation with chained equations and to validate our results a complete case analysis was performed, which showed similar results. In addition, the proportion of women with IA or OA, but not FM, in our study is higher compared with the general population [46–49]. It is known that men are often underrepresented in lifestyle intervention studies, therefore, future lifestyle studies should focus on how to attract more men [50]. Finally, no data were available on disease activity in patients with IA. Hence, future research is needed to evaluate the effect of our lifestyle intervention programme on disease activity or inflammation in IA patients.

In conclusion, a 3-month online, interactive, multifactorial lifestyle intervention programme has a positive long-term effect, even after 2 years of follow-up, on health risk and various PRO domains in patients with an IA, OA and FM.

## Supplementary Material

keae696_Supplementary_Data

## Data Availability

The data are available on request.

## Notes

*Consent to participate*: Informed consent was obtained from all individual study participants.
