# Supplementary material for: The impact of an online, lifestyle intervention programme on the lives of patients with a rheumatic and musculoskeletal disease: a pilot study
Source: Rheumatology (Oxford). 2024 Dec 28;64(6):3309–18. doi: 10.1093/rheumatology/keae696 (PMC12107065; doi:10.1093/rheumatology/keae696)
Supplement: keae696_Supplementary_Data [file keae696_supplementary_data.docx]

Supplementary material

Supplementary data S1

*Sensitivity analysis*

Two sensitivity analyses were performed: a complete case analysis and an analysis among patients with ≥5% weight loss after the intensive part of the online, interactive lifestyle intervention program.

A total of 93 patients were included in the complete case analysis, of whom 37 (43%), were diagnosed with IA, 39 (37%) with OA and 17 (24%) with FM.

Changes in health risk and PROMs over time, stratified for IA, OA, and FM, are shown in Supplementary Figures S2 and S3. Again, the greatest decrease in health risk occurred in the intensive part of the online, interactive lifestyle intervention program. Although the outcomes are less pronounced than in the original analysis, the improvement in health risks during the first 3 months were still significant, except for the improvement of waist circumference in FM patients (Supplementary Table S2).

The outcomes of the different PROMs show a similar trend. Significant PROM improvements during the intensive part of the intervention program, were found for sleep disturbance (-7.2 (1.7), p=0.006) in IA patients, fatigue (-18 (4), p=0.003) and EQ-5D-5L (0.1 (0.0), p=0.039) in OA patients. The improvement of sleep disturbance in IA patients also exceeded the MCID. Significant PROM improvements over the total follow-up period of 24 months were only found for OA patients for morning stiffness severity (-2 (0), p=0.012), fatigue (-19 (4), p=0), HAQ (-0.2 (0.1), p=0.045), EQ-5D-5L (0.1 (0.0), p=0.003) and perceived stress (-4.0 (1.0), p=0.006).

A total of 109 patients had ≥5% weight loss after the intensive part of the online, interactive lifestyle intervention program. This group consisted of 31 (35%) patients diagnosed with IA, 46 (44%) with OA and 32 (45%) with FM.

The improvements in health risks and PROMs during the intensive part of the online lifestyle intervention program were more explicit for this group of patients and the outcomes remained more stable during the aftercare period compared to the original analysis (Supplementary figure S4 and S5). The improvements of all health risks (weight, BMI and waist circumference) in all 3 diagnosis groups during the first 3 months and over the entire follow-up were significant, except for waist circumference for IA patients over the entire follow-up of 24 months (Supplementary Table S3).

Significant PROM improvements in IA patients were found for morning stiffness severity after 3 months (-2 (0), p=0) and over the entire follow-up of 24 months (-2 (0), p=0.006), sleep disturbance after 3 months (-9.9 (2.4), p=0.009) and impact on life over 24 months (-1.5 (0.4), p=0.042). In OA patients significant PROM improvements were found for fatigue after 3 and 24 months (-14 (4), p=0.012) and (-14 (4), p=0.021), EQ-5D-5L after 3 months (0.1 (0.0), p=0.009), perceived stress after 3 months (-2.9 (0.8), p=0.015) and impact on life after 3 months (-1.3 (0.3), p=0.012). Finally, significant PROM improvements for FM patients were found for morning stiffness severity after 3 months (-2 (0), p=0.048) and impact on life after 3 months (-1.5 (0.4), p=0.036). All aforementioned improvements in the IA patient group also exceeded the MCID.

Supplementary Table S1: ICHOM domains with corresponding PROMs and MCID

| **Outcome domain** | **PROM*** | **MCID**** |
| --- | --- | --- |
| Pain | Joint pain (VAS, 0-100mm) | ≥ 10 |
| Activity limitations | Morning stiffness severity (NRS, 0-10) | ≥ 1 |
|  | HAQ (0 – 3) | ≥ 0.22 |
| Fatigue | Fatigue (VAS, 0-100mm) | ≥ 10 |
| Health impact | Quality of life (EQ-5D-5L, 0 – 1) | ≥ 0.04 |
|  | Perceived stress (PSS, 0-40) | ≥ 11 |
|  | Sleep disturbance (SPI-II, 0-100) | ≥ 6 |
|  | Impact on life (NRS, 0-10) | ≥ 1 |

*PROM, Patient Reported Outcome Measurement.
**MCID, Minimal Clinically Important Difference.
*Abbreviations: EQ-5D-5L, European quality of life with-5 dimensions with 5 levels; HAQ, Health Assessment Questionnaire; mm, millimeters; MS, morning stiffness;* *NRS, numeric rating scale; PSS, Perceived Stress Scale; SPI-II, Sleep Problems Index II; VAS, Visual Analogue Score.*

Supplementary Figure S1: Flowchart.

**
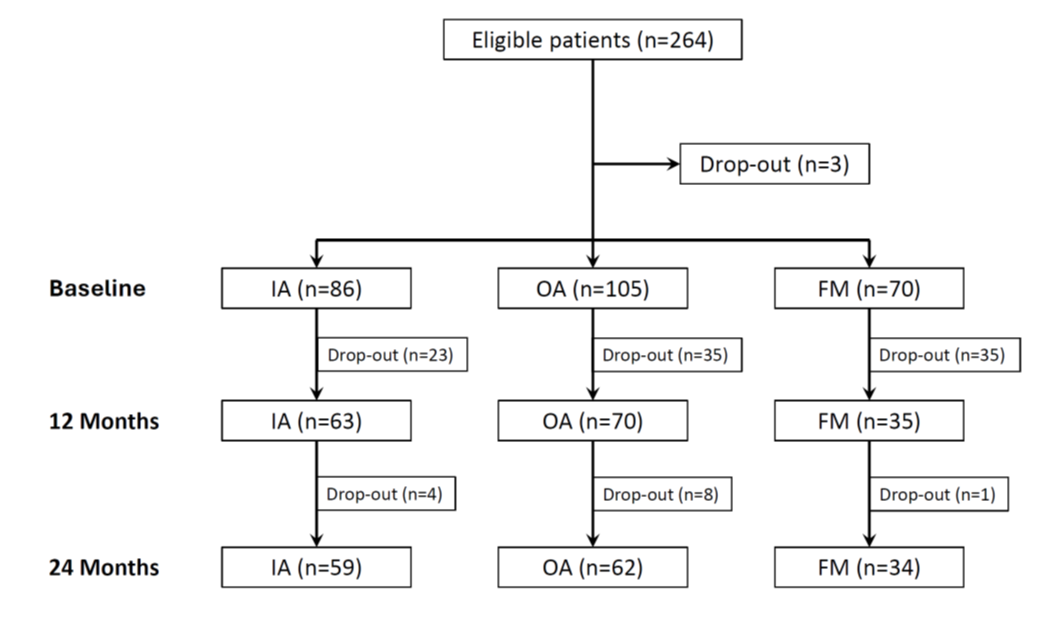
**

*Abbreviations: FM, FibroMyalgia; IA, Inflammatory Arthritis, including rheumatoid arthritis, psoriatic arthritis, spondyloarthritis or juvenile idiopathic arthritis; OA, OsteoArthritis.*

Supplementary Figure S2: Health risk over time stratified per diagnose group for complete cases.


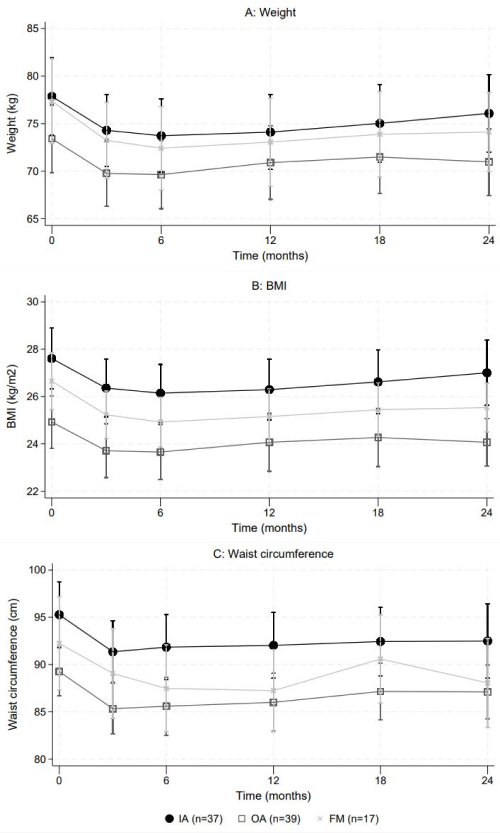


*Abbreviations: BMI, Body Mass Index; cm, centimeter; FM, FibroMyalgia; IA, Inflammatory Arthritis, including rheumatoid arthritis, psoriatic arthritis, spondyloarthritis or juvenile idiopathic arthritis; kg, kilogram; OA, OsteoArthritis.*

Supplementary Figure S3: PROMs over time stratified per diagnose group for complete cases.

**
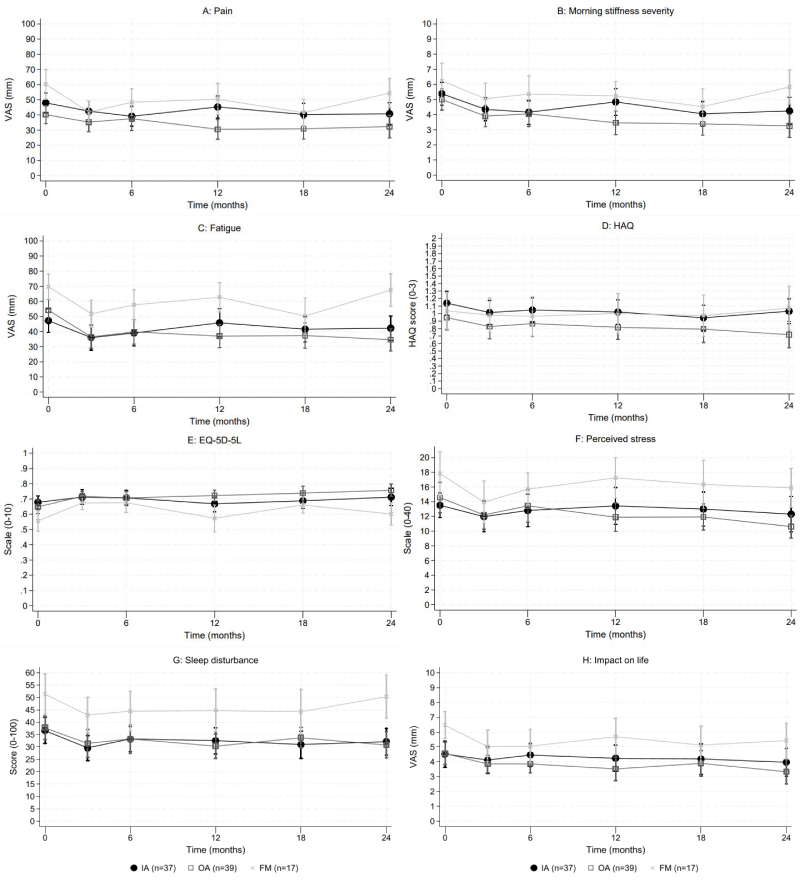
**

All figures show the mean with corresponding 95% confidence interval for the respective PROM.

*Abbreviations: EQ-5D-5L, European quality of life with 5 dimensions with 5 levels; FM, FibroMyalgia; HAQ, Health Assessment Questionnaire; IA, Inflammatory Arthritis, including rheumatoid arthritis, psoriatic arthritis, spondyloarthritis or juvenile idiopathic arthritis; OA, OsteoArthritis; PROMs, Patient Reported Outcome Measure; VAS,* *Visual Analogue Score.*

Supplementary Table S2: Health risk and PROM changes during the intensive part and aftercare period of the online, interactive, multifactorial lifestyle intervention program stratified per patient group for complete cases.

|  | IA (n=37) | | | OA (n=39) | | | | FM (n=17) | | | |  |
| --- | --- | --- | --- | --- | --- | --- | --- | --- | --- | --- | --- | --- |
|  | **0-3**  **Months*** | **3-24 Months*** | **0-24 Months*** | | **0-3**  **Months*** | **3-24**  **Months*** | **0-24**  **Months*** | | **0-3**  **Months*** | **3-24 Months*** | **0-24**  **Months*** | |
| Health risks |  |  |  | |  |  |  | |  |  |  | |
| Weight (kg) | -3.6 (0.5)* | 1.8 (0.7) | -1.8 (0.8) | | -3.6 (0.4)* | 1.2 (0.9) | -2.4 (0.9) | | -4.1 (0.9)ⁱ | 0.9 (0.5) | -3.2 (1.2) | |
| BMI (kg/m^2^) | -1.3 (0.2)* | 0.7 (0.3) | -0.6 (0.3) | | -1.2 (0.1)* | 0.4 (0.3) | -0.9 (0.3) | | -1.4 (0.3)⁰ | 0.3 (0.2) | -1.1 (0.4) | |
| Waist circumference(cm) | -3.9 (0.8)* | 1.1 (1.0) | -2.8 (1.3) | | -3.9 (0.7)* | 1.8 (1.1) | -2.2 (1.1) | | -3.2 (1.2) | - 1.0 (1.1) | -4.2 (1.2) | |
| PROMs |  |  |  | |  |  |  | |  |  |  | |
| Pain (VAS 0-100) | -6 (3) | -2 (4) | -7 (4) | | -5 (4) | -3 (4) | -8 (5) | | -18 (6) | 12 (6) | -6 (6) | |
| MS severity (NRS 0-10) | **-1 (0)** | -0 (0) | **-1 (0)** | | -1 (0) | -1 (0) | -2 (0)⁰ | | -1 (1) | 1 (1) | -0 (1) | |
| Fatigue (VAS 0-100) | **-11 (4)** | 6 (4) | -5 (4) | | -18 (4)ⁱ | -2 (4) | -19 (4)* | | -18 (7) | 16 (4)⁰ | -2 (7) | |
| HAQ (0-3) | -0.1 (0.1) | 0.0 (0.1) | -0.1 (0.1) | | -0.1 (0.1) | -0.1 (0.1) | -0.2 (0.1)⁰ | | -0.1 (0.1) | 0.1 (0.1) | 0.0 (0.1) | |
| EQ-5D-5L (0-1) | 0.0 (0.0) | -0.0 (0.0) | 0.0 (0.0) | | 0.1 (0.0)⁰ | 0.0 (0.0) | 0.1 (0.0)ⁱ | | 0.1 (0.0) | -0.1(0.0) | 0.1 (0.0) | |
| Perceived stress (PSS 0-40) | -1.5 (0.7) | 0.2 (0.7) | -1.3 (1.1) | | -2.4 (0.9) | -1.6 (0.9) | -4.0 (1.0)ⁱ | | -3.9 (1.5) | 1.9 (1.5) | -1.9 (1.6) | |
| Sleep disturbance (SPI-II 0-100) | **-7.2 (1.7)ⁱ** | 2.5 (1.8) | -4.3 (2.4) | | -6.3 (2.6) | -0.6 (2.2) | -7.0 (2.5) | | -8.5 (3.0) | 7.5 (3.7) | -1.1 (3.2) | |
| Impact on life (NRS 0-10) | -0.4 (0.5) | -0.3 (0.4) | -0.7 (0.5) | | -0.7 (0.5) | -0.5 (0.6) | -1.2 (0.6) | | -1.5 (0.6) | 0.4 (0.8) | -1.1 (0.6) | |

* Statistical comparisons were performed between baseline and 3 months, 3 and 24 months and baseline and 24 months, using a paired t-test.

For each variable mean (sd) and statistical significance are reported.

⁰p<0,05

ⁱP<0.01

*p<0,001

Clinically relevant improvements compared to MCID are shown in **bold.**

*Abbreviations: BMI, Body Mass Index; cm, centimeter; EQ-5D-5L, European quality of life with-5 dimensions with 5 levels; FM, FibroMyalgia; HAQ, Health Assessment Questionnaire; IA, Inflammatory Arthritis, including rheumatoid arthritis, psoriatic arthritis, spondyloarthritis or juvenile idiopathic arthritis; kg, kilogram; MCID, minimal clinical important difference; MS, morning stiffness; NRS, numeric rating scale; OA, OsteoArthritis; PROM, Patient Reported Outcome Measure; PSS, Perceived Stress Scale; sd, standard deviation; SPI-II, Sleep Problems Index II; VAS,* *Visual Analogue Score.*

Supplementary Figure S4: Health risk over time stratified per diagnose group for patients with ≥5% weight loss.


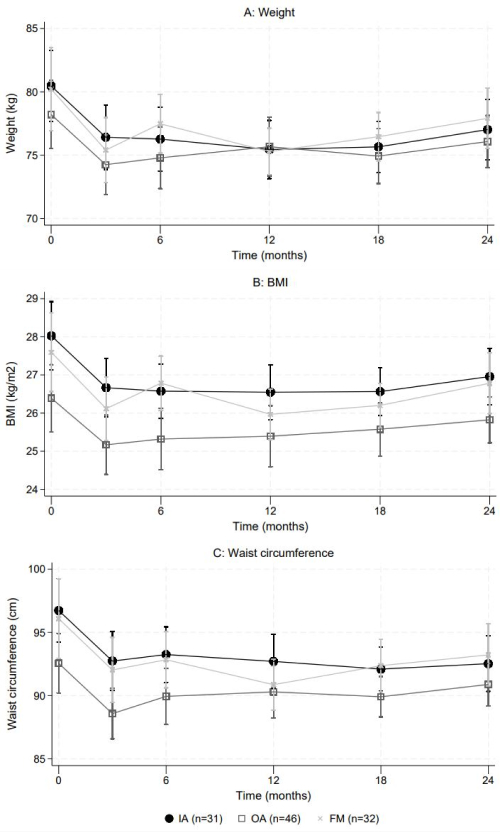


*Abbreviations: BMI, Body Mass Index; cm, centimeter; FM, FibroMyalgia; IA, Inflammatory Arthritis, including rheumatoid arthritis, psoriatic arthritis, spondyloarthritis or juvenile idiopathic arthritis; kg, kilogram; OA, OsteoArthritis.*

Supplementary Figure S5: PROMs over time stratified per diagnose group for patients with ≥5% weight loss.

**
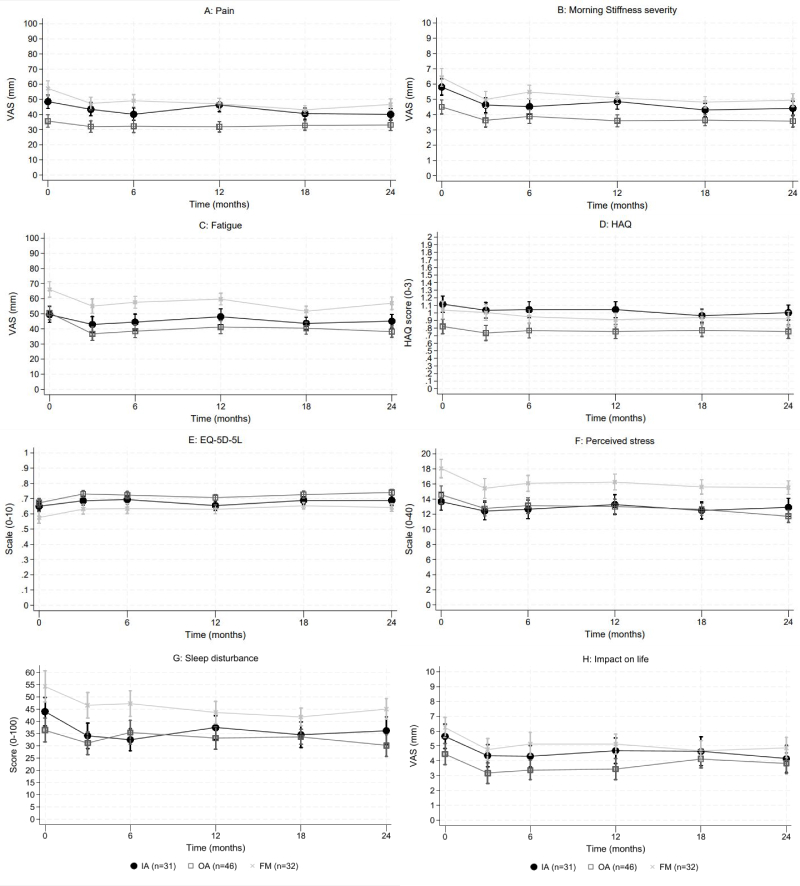
**

All figures show the mean with corresponding 95% confidence interval for the respective PROM.

*Abbreviations: EQ-5D-5L, European quality of life with 5 dimensions with 5 levels; FM, FibroMyalgia; HAQ, Health Assessment Questionnaire; IA, Inflammatory Arthritis, including rheumatoid arthritis, psoriatic arthritis, spondyloarthritis or juvenile idiopathic arthritis; OA, OsteoArthritis; PROMs, Patient Reported Outcome Measure; VAS,* *Visual Analogue Score.*

Supplementary Table S3: Health risk and PROM changes during the intensive part and aftercare period of the online, interactive, multifactorial lifestyle intervention program stratified per patient group for patients with ≥5% weight loss.

|  | IA (n=31) | | | OA (n=46) | | | | FM (n=32) | | | |  |
| --- | --- | --- | --- | --- | --- | --- | --- | --- | --- | --- | --- | --- |
|  | **0-3**  **Months*** | **3-24 Months*** | **0-24 Months*** | | **0-3**  **Months*** | **3-24**  **Months*** | **0-24**  **Months*** | | **0-3**  **Months*** | **3-24 Months*** | **0-24**  **Months*** | |
| Health risks |  |  |  | |  |  |  | |  |  |  | |
| Weight (kg) | -9.3 (1.3)* | 1.9 (0.7) | -7.4 (1.7)ⁱ | | -8.1 (1.1)* | 0.8 (1.0) | -7.3 (1.5)* | | -10.0 (1.4)* | 2.9 (2.2) | -7.2 (2.1)⁰ | |
| BMI (kg/m^2^) | -3.1 (0.4)* | 0.6 (0.2) | -2.4 (0.6)ⁱ | | -2.5 (0.3)* | 0.4 (0.3) | -2.1 ((0.5)* | | -3.2 (0.4)* | 0.8 (0.5) | -2.4 (0.5)ⁱ | |
| Waist circumference(cm) | -6.6 (1.4)* | 0.4 (1.1) | -6.2 (1.8) | | -7.9 (1.2)* | 1.5 (1.) | -6.5 (1.5)ⁱ | | -8.0 (1.3)* | 1.5 (1.8) | -6.5 (1.7)ⁱ | |
| PROMs |  |  |  | |  |  |  | |  |  |  | |
| Pain (VAS 0-100) | **-12 (4)** | -0 (4) | **-12 (4)** | | -9 (3) | 4 (4) | -6 (4) | | -14 (5) | 1 (4) | -14 (4) | |
| MS severity (NRS 0-10) | **-2 (0)*** | 0 (0) | **-2 (0)ⁱ** | | -1 (3) | -0 (0) | -1 (0) | | -2 (0)⁰ | 0 (0) | -1 (1) | |
| Fatigue (VAS 0-100) | **-14 (5)** | 5 (4) | -9 (5) | | -14 (4)⁰ | 1 (4) | -14 (4)ⁱ | | -18 (6) | 3 (3) | -15 (5) | |
| HAQ (0-3) | -0.1 (0.1) | 0.0 (0.1) | **-0.1 (0.1)** | | -0.2 (0.1) | 0.0 (0.1) | -0.1 (0.1) | | 0.0 (0.1) | -0.2 (0.1) | -0.1 (0.1) | |
| EQ-5D-5L (0-1) | **0.1 (0.0)** | -0.0 (0.0) | 0.1 (0.0) | | 0.1 (0.0)ⁱ | -0.0 (0.0) | 0.1 (0.0) | | 0.1 (0.0) | 0.0 (0.0) | 0.1 (0.0) | |
| Perceived stress (PSS 0-40) | -2.6 (1.0) | -0.7 (0.7) | -3.3 (1.1) | | -2.9 (0.8)⁰ | -0.0 (0.8) | -2.9 (0.9) | | -2.4 (1.0) | -0.0 (1.2) | -2.4 (1.0) | |
| Sleep disturbance (SPI-II 0-100) | **-9.9 (2.4)ⁱ** | 2.1 (1.8) | **-7.8 (2.6)** | | -5.4 (1.7) | -0.7 (1.6) | -5.5 (2.3) | | -7.8 (3.1) | -1.5 (3.2) | -9.3 (3.2) | |
| Impact on life (NRS 0-10) | **-1.3 (0.5)** | -0.2 (0.4) | **-1.5 (0.4)⁰** | | -1.3 (0.3)⁰ | 0.6 (0.5) | -0.5 (0.5) | | -1.5 (0.4)⁰ | 0.1 (0.5) | -1.4 (0.4) | |

* Statistical comparisons were performed between baseline and 3 months, 3 and 24 months and baseline and 24 months, using a paired t-test.

For each variable mean (sd) and statistical significance are reported.

⁰p<0,05

ⁱP<0.01

*p<0,001

Clinically relevant improvements compared to MCID are shown in **bold.**

*Abbreviations: BMI, Body Mass Index; cm, centimeter; EQ-5D-5L, European quality of life with-5 dimensions with 5 levels; FM, FibroMyalgia; HAQ, Health Assessment Questionnaire; IA, Inflammatory Arthritis, including rheumatoid arthritis, psoriatic arthritis, spondyloarthritis or juvenile idiopathic arthritis; kg, kilogram; MCID, minimal clinical important difference; MS, morning stiffness; NRS, numeric rating scale; OA, OsteoArthritis; PROM, Patient Reported Outcome Measure; PSS, Perceived Stress Scale; sd, standard deviation; SPI-II, Sleep Problems Index II; VAS,* *Visual Analogue Score.*
